# Cement augmentation of the proximal femoral nail antirotation for the treatment of two intertrochanteric fractures - a comparative finite element study

**DOI:** 10.1186/s12891-021-04878-2

**Published:** 2021-12-02

**Authors:** Liqin Zheng, Xinmin Chen, Yongze Zheng, Xingpeng He, Jingxiong Wu, Ziling Lin

**Affiliations:** 1 The First Clinical Medical College, Guangzhou University of Chinese Medicine, Guangzhou, Guangdong China; 2 Department of Orthopedic, Zhongxiang People’s Hospital, Jingmen, Hubei China; 3 Department of Orthopedic, Puning Hospital of Traditional Chinese Medicine, Jieyang, Guangdong China; 4 Department of Osteoarticular Surgery, Integrated Traditional Chinese and Western Medicine Hospital of Pengjiang District of Jiangmen City, Jiangmen, Guangdong China; 5 Department of Orthopedic, The First Affiliated Hospital of Guangzhou University of Chinese Medicine, Guangzhou, Guangdong China

**Keywords:** Bone cement augmentation, Proximal femoral nail antirotation, Intertrochanteric fracture, Finite element analysis

## Abstract

**Background:**

There are concerns regarding initial stability and cutout effect in proximal femoral nail antirotation (PFNA) treating intertrochanteric fractures. No study have used finite element analysis (FEA) to investigate the biomechanics. This study aimed to compare the cutout effect, stress and displacement between stable (AO31-A1.3) and unstable (AO31-A2.2) intertrochanteric fractures treated by cement augmented PFNA.

**Methods:**

Four femoral finite element models (FEMs) were constructed and tested under the maximum loading during walking. Non-augmented and augmented PFNA in two different intertrochanteric fractures were respectively simulated, assuming Tip Apex Distance (TAD) < 25 mm within each FEM. The cutout effect, stress and displacement between femur and PFNA were compared in each condition.

**Results:**

Cutout effect was observed in both non-augmented femoral head and was more apparently in unstable intertrochanteric fracture model. After reinforced by bone cement, no cutout effect occurred in two models. Stress concentration were observed on medial part of intertrochanteric region and the proximal part of helical blade before augmented while were observed on femoral shaft and the conjunction between blade and nail after augmented in both FEMs. Displacement mainly appeared on femoral head and the helical blade tip before augmented while distributed moderately on intertrochanteric region and the upper part of nail after augmented in both FEMs. The maximum stress and displacement value of femur decreased both in stable and unstable model after augmented but was more significantly in the unstable one. The maximum stress and displacement value of PFNA increased both in stable and unstable model after augmented but was more significantly in the unstable one.

**Conclusion:**

Our FEA study indicated that the cement augmentation of the PFNA biomechanically enhances the cutout resistance in intertrochanteric fracture, this procedure is especially efficient for the unstable intertrochanteric fracture.

## Introduction

Proximal femoral nail antirotation (PFNA) has a better clinical effect than other internal fixators in treating intertrochanteric fractures, especially the unstable type [1, 2]. However, as the number of cases increased, so did the incidence of complications related to internal fixation (i.e. thigh pain, leg shortening, and cutout, even cut through occur occasionally with rehabilitation). Cutout is one of the common complications, with an incidence of up to 3-15% as reported in literatures [3].

The application of bone cement (Polymethylmethacrylate, PMMA) could potentially reduce the risk of cutout complications and reoperations. Biomechanically, a clear benefit of bone cement augmentation has been demonstrated as exemplified by PFNA placing helical blade and later-injected cement volume [4, 5]. Clinically, even with a higher Tip Apex Distance (TAD) and Calcar referenced Tip Apex Distance (Cal-TAD), the augmentation of PFNA blade have a superiority to prevent reoperations by strengthening the osteosynthesis construct to resist symptomatic implant migration [6]. Further, it is reported that there was a low risk of negative biological side effects of cement augmentation (i.e. pressure-induced avascular necrosis) [7, 8].

The concept of lateral wall thickness has been applied to clinical classification and procedures. Lateral wall thickness is defined as the distance in millimeters (mm) from a reference point 3 cm below the innominate tubercle of the great trochanter angle 135° upward to the fracture line on the anteroposterior X-ray [9, 10]. In the AO/OTA (2018) classification, the lateral wall thickness ≤ 20.5 mm is adopted to define whether the lateral wall is complete and whether the fracture is stable [11]. In previous studies on bone cement augmented PFNA treating intertrochanteric fractures, the influence of lateral wall thickness under the updated classification (AO/OTA (2018)) has not been explored. The biomechanical efficiency of cement augmented PFNA treating intertrochanteric fracture under AO/OTA (2018) classification remain unclear.

The objective of this study was to use finite element analysis (FEA) to investigate the stable (AO31-A1.3 with lateral wall thickness > 20.5 mm) and unstable (AO31-A2.2 with lateral wall thickness < 20.5 mm) intertrochanteric fracture, under AO/OTA (2018) classification, treated by cement augmented PFNA based on the following criteria:

- A varus collapse of 2° of head-neck fragment in coronal plane, indicative for loosening of the helical blade, was defined as the point of failure [5];
- Cutout effect of helical blade when femoral head varus collapse;
- Stress and displacement distribution on femoral and PFNA when femoral head varus collapse.

To simulate cutout deformity, we utilized the element deletion method which is used to model material failure [12, 13]. When strain exceeds failure strain, elements will be deleted and the remaining defect will lead to further failure propagation. This method may be appropriate in analyzing the mechanism of cutout.

## Materials and methods

### Finite element model

**Fig. 1:**
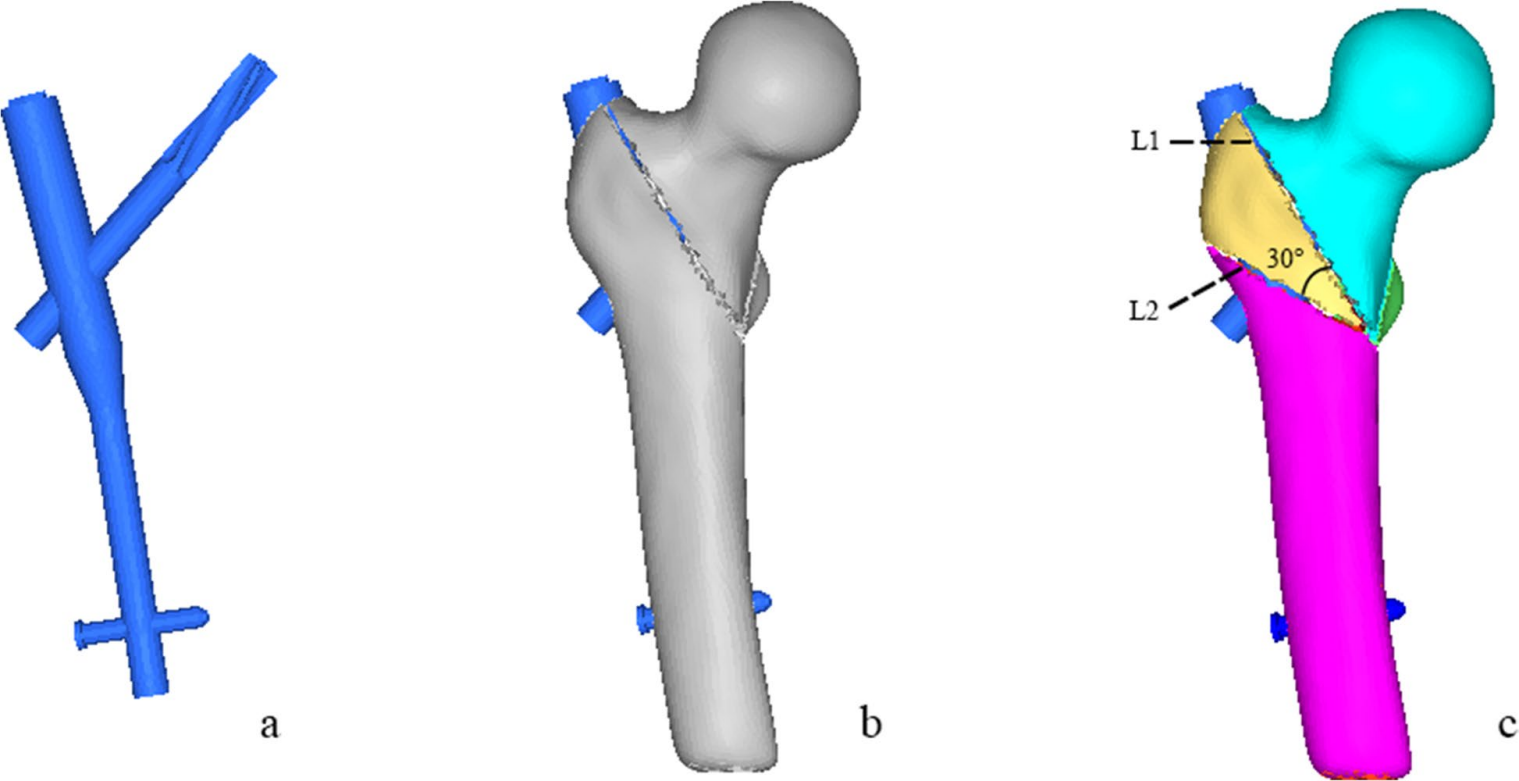
FE model: **a**. PFNA; **b**.AO31-A1.3 fracture with PFNA internal fixation; **c**. AO31-A2.2 fracture with PFNA internal fixation

The CT data of a patient with hip fracture (female, 71 years old, 165 cm height, and 48 kg weight) were obtained and imported into Mimics 19.0 (Materialise Inc., Leuven, Belgium) in DICOM format. A three-dimension (3D) proximal femora model was preliminarily established through region growth, cavity filling, editing mask, wrapping and smoothing. Surface optimization was performed on the above model using Geomagic Studio 2013 (GEOMAGIC, USA) to obtain an optimized 3D femur. The components of PFNA (Dabo Medical Devices Co., Ltd., Xiamen, China) was designed and assembled in SolidWorks (Dassault Systemes, USA). The length of nail was 200 mm with a diameter of proximal and distal end 16 mm and 10 mm, respectively; the length of helical blade was 107 mm with a diameter 10 mm (Fig. 1a). Then, the optimized 3D model of femur was continued to be imported in SolidWorks. According to the clinical operation techniques, the PFNA was fixed to the proximal femur and established the TAD < 25 mm. In Hypermesh14.0 software (Altair, USA), the assembled model was meshed as tetrahedral with size of 2 mm [14]. Three millilitre bone cement was considered as a safe injection dose [4], accordingly, a certain number of tetrahedral elements around the helical blade were organized as bone cement base on the tetrahedral size. Using organization tool in Hypermesh14.0 software, the proximal femur was organized as a mixed model containing trabecular bone and a 2-mm cortical bone layer [15]. AO31-A2.1 is a default subtype in AO/OTA (2018) classification, in this study, AO31-A1.3 and AO31-A2.2 fractures were taken as the representative of the stable and unstable type, because these two type of fractures account for the major proportion of intertrochanteric fractures. The fracture line was performed in Hypermesh14.0 software as following steps:

- Rotate the proximal femur along the vertical axis to fully display the lesser trochanter. An origin point was taken as the demarcation of the middle 1/3 of the line between the apex of lesser trochanter and the contralateral cortical bone. A straight line was led from the upper and lower bases of the lesser trochanter to the point, and the area enclosed by the two lines was the osteotomy area of the lesser trochanter.
- On anteroposterior position, a straight line was made from the apex of greater trochanter to the base of the lesser trochanter, which was used as the osteotomy line L1. The thickness of the lateral wall at this condition was defined as T1, and this model was regard as AO31-A1.3. As shown in Fig. 1b;
- On anteroposterior position, based on AO31-A1.3 model above, taking the base of lesser trochanter as origin point, an osteotomy line L2 was led to the lateral of the greater trochanter, so that the angle between L2 and L1 was 30°. The thickness of the lateral wall at this condition was defined as T2, and this model was regard as AO31-A2.2. As shown in Fig. 1c.

The lateral wall thicknesses of two intertrochanteric fracture models are shown in Table 1. The numbers of elements and nodes of the FEA models are shown in Table 2.

**Table 1:** The lateral wall thicknesses of two intertrochanteric fracture models

AO classification	Lateral wall thickness (mm)
31-A1.3	27.63
31-A2.2	17.34

**Table 2:** Numbers of elements and nodes of FEA models

	AO31-A1.3		AO31-A2.2	
	Element	Node	Element	Node
Femoral	86,599	20,024	85,428	20,003
PFNA	16,880	5017	16,880	5017
Bone cement	3183	1028	3183	1028

### Material properties and contact surfaces

We utilized the element deletion method to simulate helical blade cutout deformity, accordingly, the material model MAT_03 in Hypermesh14.0 software was adopted to simulate bone and implant behavior where their fracture or failure was defined by specifying a plastic failure strain and initiated and propagated by element deletion [12, 16]. Strain rate effect was included in the models. PFNA implants were made of the high-strength titanium alloy TiAl6V4. For a volume BMD density (ρBMD) less than 250 mg/cm^3^, there is a high risk of fixation failure after surgical treatment of intertrochanteric fractures [17], therefore, in this study, in order to successfully demonstrate the cutout deformity, ρBMD was set to 200 mg/cm^3^. According to the equations between ρBMD and elastic modulus (E) and yield stress (σ) of trabecular bone, see Eq. (1) [18], Eq. (2) [19], Eq. (3) [20], Eq. (4) [21], the apparent density (ρ_app_), E and σ of trabecular bone were calculated.

1 $${\uprho}_{\mathrm{ash}}=79.8+0.79{2\uprho}_{\mathrm{BMD}}\ \left(\mathrm{mg}/{\mathrm{cm}}^3\right)$$

2 $$\uprho \mathrm{app}=\frac{\boldsymbol{\uprho} \mathbf{ash}}{\mathbf{0.55}}\ \left(\mathrm{g}/\mathrm{cm}3\right)$$

3 $$\mathrm{E}=1.310\ {\uprho}_{\mathrm{app}}^{1.40}\ \left(\mathrm{Gpa}\right)$$

4 $$\upsigma =0.0062\mathrm{E}-0.41\ \left(\mathrm{MPa}\right)$$

According to the equations between cortical bone ρ_app_, E and σ, see Eq. (5) [22], Eq. (6) [23], cortical bone’s E and σ were calculated. In this study, the median ρapp was 1.525 g/cm^3^ according to Eq. (5) was chosen. The material contents of PFNA and bone cement were acquired from literatures. See Table 3.

5 $$\mathrm{E}=-13.43+14.26{1\uprho}_{\mathrm{app}}\ \left(\mathrm{Gpa}\right)$$

6 $$\upsigma =6.01\mathrm{E}+35.76\ \left(\mathrm{Mpa}\right)$$

**Table 3:** Material contents of femoral, PFNA and bone cement

	Trabecular	Cortical	PFNA [24]	Bone cement [24]
Apparent density(ρ_app_), g/cm^3^	0.433	1.525	4.5	1.18
Elastic Modulus(E), Gpa	0.406	8.318	120	220
yield stress (σ), Mpa	2.107	85.751	1086	100
Poisson’s ratio (v)	0.3	0.3	0.31	0.20
Hardening parameter (β)^a^	0.1 [25]		0.1 [26]	NR^c^
C^b^	2.5 [25]		80,000 [27]	NR
P^b^	7 [25]		1.1 [27]	NR
Failure strain (%)	0.7 [21]	0.9 [28]	22 [29]	1.3 [30]

^a^The material model MAT_03 allows for a hardening parameter β, where 0 ≤ β ≤ 1

^b^Parameter of strain rate effect in Cowper–Symonds model

^c^Not reported in literatures

Friction contacts were established between the components of PFNA, between fracture fragments, between fracture fragments and PFNA, and between helical blade and bone cement. The friction coefficient between the components of PFNA, between fracture fragments were set to 0.23 and 0.46, respectively [31]; the coefficients between PFNA and femur, between helical blade and bone cement were set to 0.3 and 0.2, respectively [32].

### Boundary and loading conditions

**Fig. 2:**
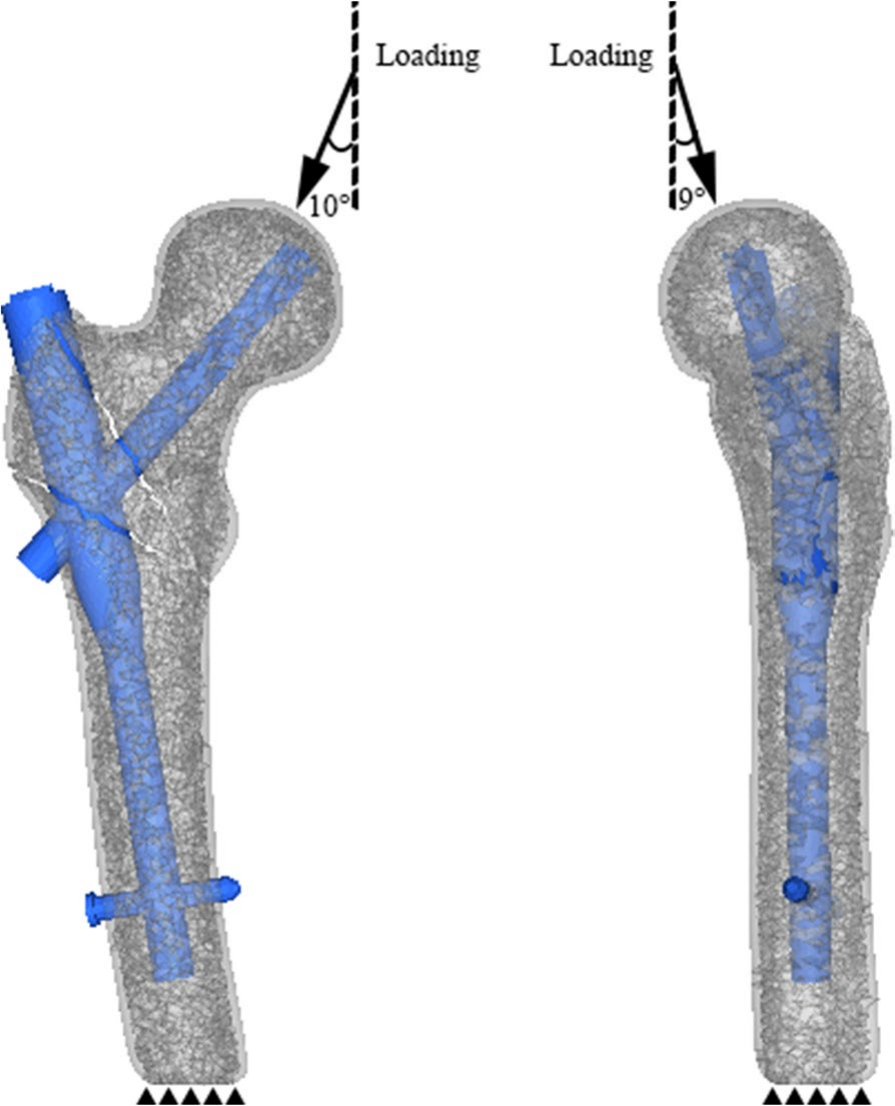
Boundary and loading conditions: An axial 2100 N net loading was applied to the proximal spherical surface of the femoral head and the femoral FEA model was fully fixed at the distal end

An axial 2100 N net loading was applied to the proximal spherical surface of the femoral head [33], and the loading was applied to the femoral head at 10° laterally in the coronal plane and 9° posteriorly on the sagittal plane [33], the femoral FEA model was fully fixed (zero displacement) at the distal end (Fig. 2).

### FEM analysis

The above FEMs were submitted to LS-DYNA (LSTC, Livermore, CA) software for simulation to investigate the biomechanical effect depending upon bone cement augmentation of PFNA while considering two different intertrochanteric fracture types. The cutout effect of helical blade in femoral head and the stress and displacement of femur and PFNA were investigated.

## Results

### Cutout effect

**Fig. 3:**
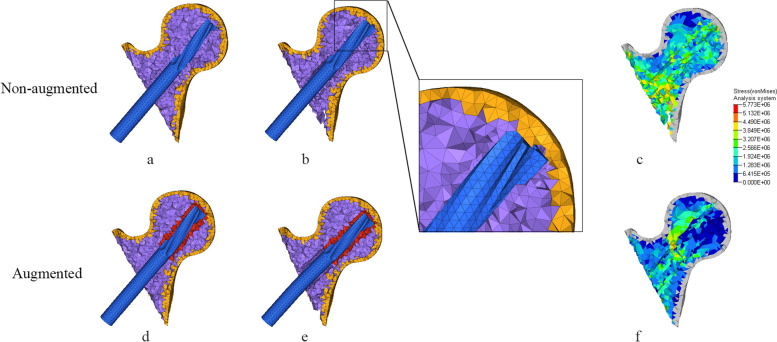
Helical blade tip location and von-mises stress (Pa) distribution in AO31-A1.3 head-neck fragment trabecular. **a** and **d**: Initial position of helical blade tip in femoral head. **b** and **e**: Location of helical blade tip when femoral head varus collapse at 2°; the non-augmented blade cutout in femoral head while remain intact in the augmented one. **c** and **f**: Von-mises stress distribution of trabecular bone when femoral head varus collapse at 2°

**Fig. 4:**
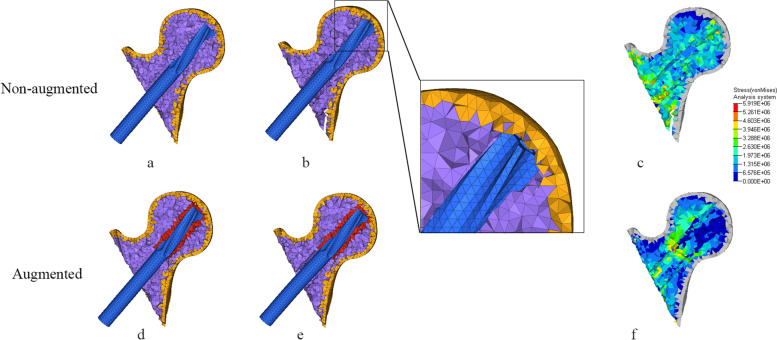
Helical blade tip location and von-mises stress (Pa) distribution in AO31-A2.2 head-neck fragment trabecular. **a** and **d**: Initial position of helical blade tip in femoral head. **b** and **e**: Location of helical blade tip when femoral head varus collapse at 2°; the non-augmented blade cutout in femoral head while remain intact in the augmented one. **c** and **f**: Von-mises stress distribution of trabecular bone when femoral head varus collapse at 2°

Figures 3 and 4, Table 5 show the cutout effect of helical blade. The non-augmented helical blade cutout in femoral head trabecular bone in both AO31-A1.3 and AO31-A2.2 fracture models. The cutting displacement of helical blade in AO31-A2.2 fracture model was higher than that in AO31-A1.3. After reinforced by bone cement, there was no relative displacement or cutting effect occurred between helical blade and femoral head in both AO31-A1.3 and AO31-A2.2 models.

### Von-Mises stress distribution on femur and PFNA

**Fig. 5:**
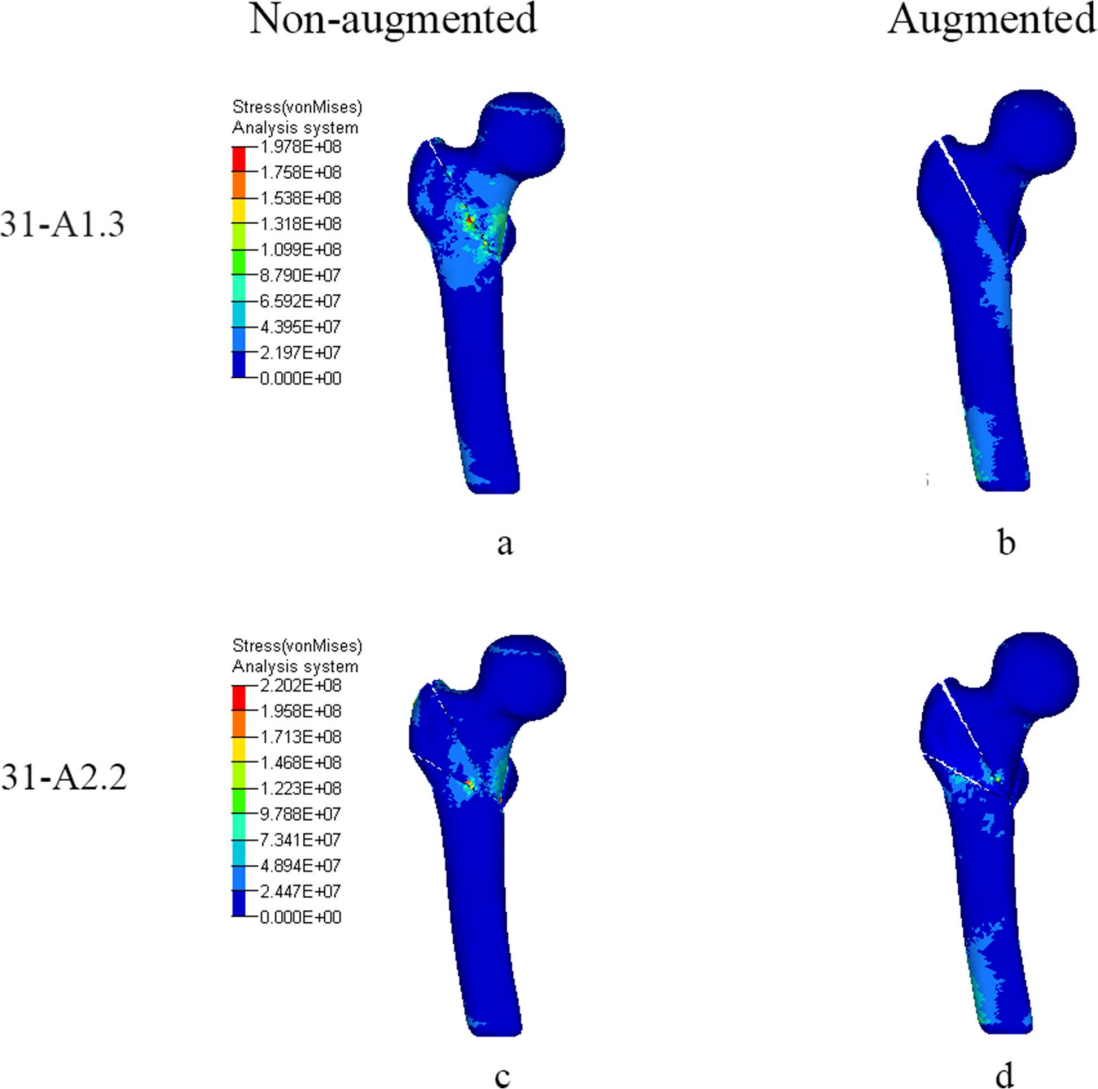
Von-mises stress (Pa) distribution on femur when femoral head varus collapse at 2°. **a** and **c**: In non-augmented model, stress was concentrated at the medial part of intertrochanteric cortical. **b** and **d**: In augmented model, stress was concentrated at the cortical below lateral locking bolt hole

**Fig. 6:**
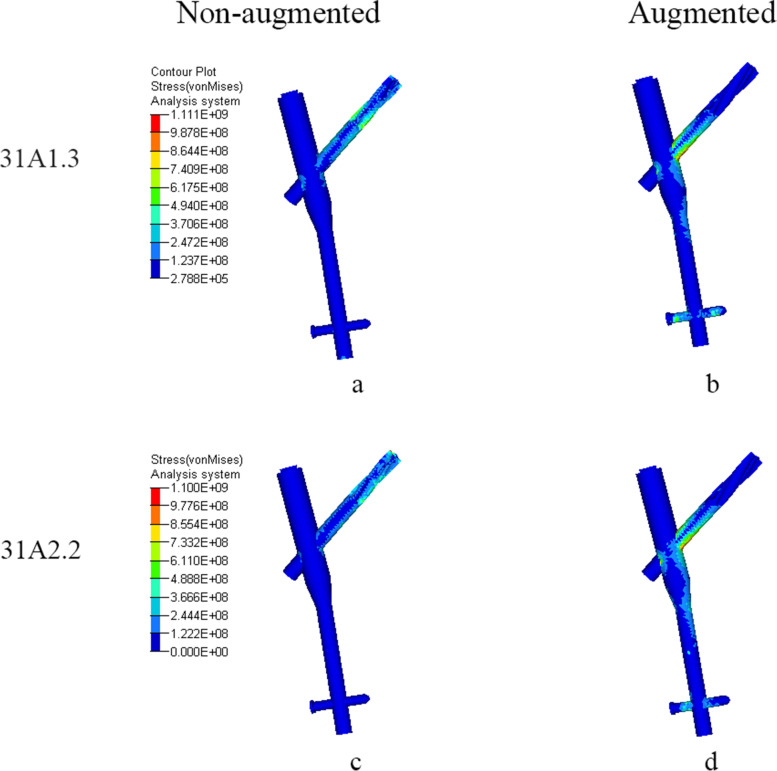
Von-mises stress (Pa) distribution on PFNA when femoral head varus collapse at 2°. **a** and **c**: In non-augmented model, stress was concentrated at the proximal part of helical blade. **b** and **d**: In augmented model, stress was concentrated at the junction between helical blade and nail

Figures 5 and 6, Table 4 show the Von-Mises stress of femur and PFNA. As for femur, before two models were strengthened, the stress of the femur was concentrated on medial part of intertrochanteric region and the stress of AO31-A2.2 was higher than that of AO31-A1.3. After reinforced by bone cement, the stress concentration of femurs both shifted from the medial part of intertrochanteric region to femoral shaft and both their maximum stress decreased, but the stress of femur of AO31-A2.2 was still higher than that of AO31-A1.3. Stress of head-neck trabecular decreased both in AO31-A1.3 and AO31-A2.2 after bone cement augmented. The decreasing rate of Von-Mises stress in AO31-A2.2 was more significantly than that in AO31-A1.3.

**Table 4:** Von-mises stress of femur and PFNA (Mpa). The changing rates of maximum Von -mises stress, compared to non-augmented, are shown in parentheses

AO classification	Status	Maximum stress of femur	Maximum stress of head-neck trabecular	Maximum stress of PFNA
31-A1.3	Non-augmented	193.4	4.5	829.4
	Augmented	177.2 (−8.4%)	4.2 (−6.7%)	1005.0 (+ 21.2%)
31-A2.2	Non-augmented	217.6	4.7	797.2
	Augmented	186.1 (−14.5%)	4.1 (−12.8%)	974.1 (+ 22.2%)

As for PFNA, before two models were strengthened, stress concentration appeared on the proximal part of helical blade and the stress of AO31-A2.2 was lower than that of AO31-A1.3. After reinforced by bone cement, the stress of PFNA both concentrated on the conjunction between blade and nail and increased significantly, however, the stress of AO31-A2.2 was still lower than that of AO31-A1.3. The increasing rate of Von-Mises stress in AO31-A2.2 was higher than that in AO31-A1.3.

### Displacement of femur and PFNA

**Fig. 7:**
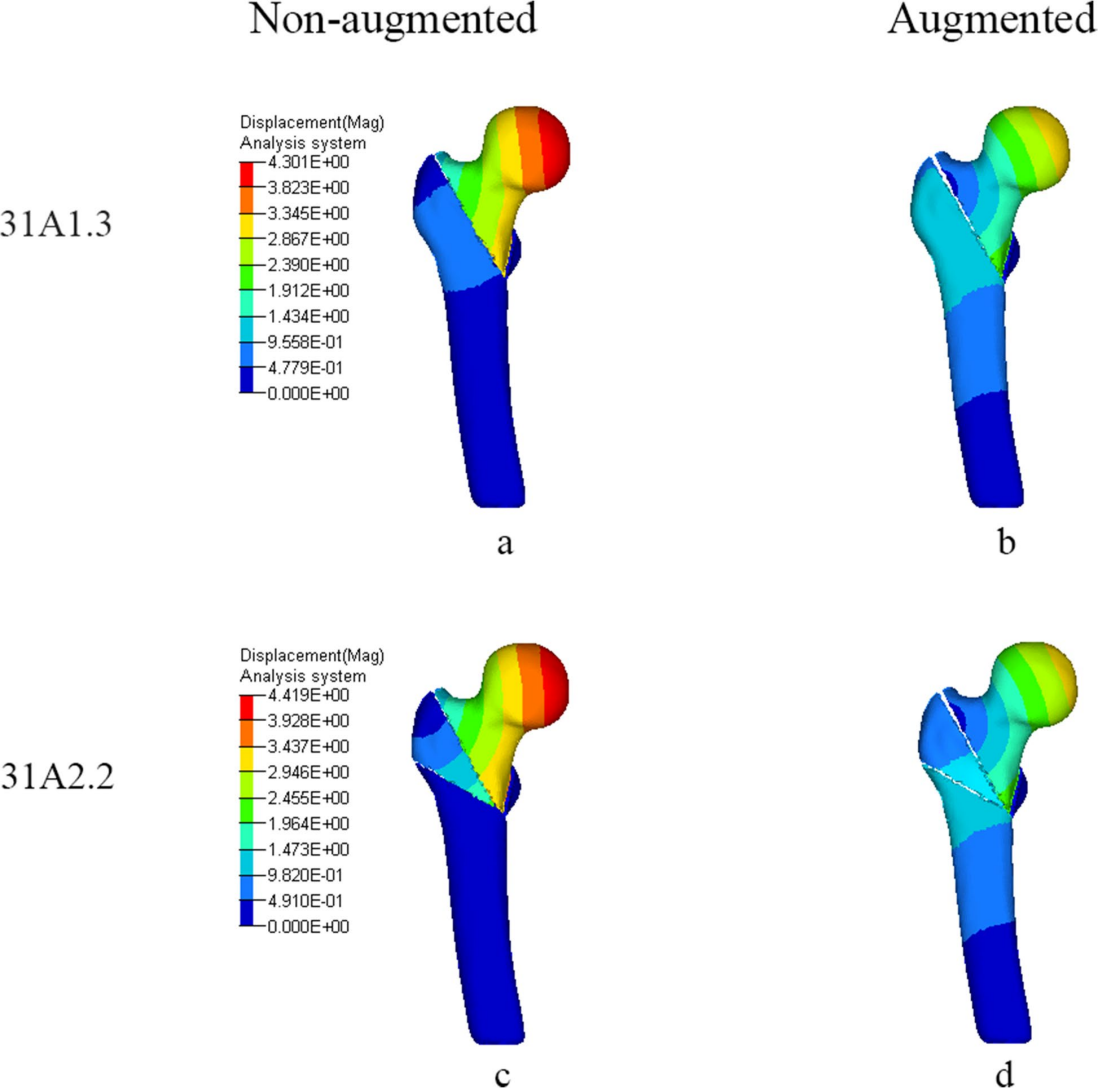
Displacement (mm) of femur when femoral head varus collapse at 2°. **a** and **c** In non-augmented model, displacement was concentrated at the medial side of femoral head. **b** and **d**: In augmented model, displacement was concentrated at the medial side of femoral head but also distributed moderately on intertrochanteric zone and femoral shaft

**Fig. 8:**
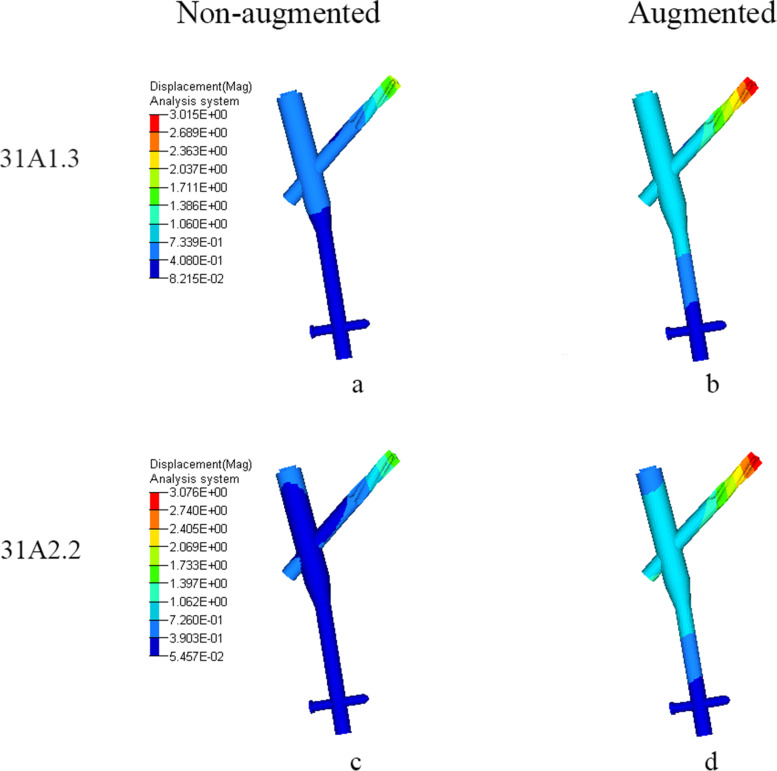
Displacement (mm) of PFNA when femoral head varus collapse at 2°. **a** and **c**: In non-augmented model, displacement concentrated at the tip of helical blade. **b** and **d**: In augmented model, displacement concentrated at the tip of helical blade and distributed moderately on the proximal part of nail

Figures 7 and 8, Table 5 show the displacement of femur and PFNA. As for femur, in two non-augmented models, the displacement primarily appeared on femoral head, a higher displacement occurred on AO31-A2.2 than that on AO31-A1.3. After strengthened by bone cement, the displacement were both concentrated at femoral head but also distributed moderately on intertrochanteric region, and their displacement decreased to an equivalence. The decreasing rate of displacement in AO31-A2.2 was higher than that in AO31-A1.3.

**Table 5:** Displacement of femur and PFNA (mm). The changing rates of maximum displacement, compared to non-augmented, are shown in parentheses

AO classification	Status	Maximum displacement of femur	Maximum displacement of PFNA	Cutting displacement of helical blade
31-A1.3	Non-augmented	4.30	1.91	2.31
	Augmented	3.26 (−24.2%)	3.05 (+ 59.7%)	N*
31-A2.2	Non-augmented	4.42	1.68	2.65
	Augmented	3.26 (−26.2%)	3.04 (+ 80.9%)	N

N*: No relative displacement between helical blade and femoral head trabecular

As for PFNA, before two models were strengthened, displacement both mainly appeared on the tip of helical blade and a higher displacement occurred on AO31-A1.3. After reinforced by bone cement, displacement both concentrated at the proximal part of helical blade and distributed partially on the upper part of nail. Their displacement nearly increased to an equivalence, but the increasing rate of displacement in AO31-A2.2 was significantly higher than that in AO31-A1.3.

## Discussion

Bone cement has been accepted for the treatments in patients with osteoporotic intertrochanteric fracture [34, 35]. Dynamic hip screws (DHS) reinforced with bone cement has a satisfying effect in reducing the total displacement of intertrochanteric fracture and enhanced the rotational stability of proximal fragment [36], promoted faster pain relief and improved fracture healing [35]. The geometry of PFNA helical blade is different from other internal fixations. Once the blade rotated in femoral head, its cutting resistance ability will be weaken and make it more likely to fail. However, the use of bone cement can broaden the bone-fixation contact interface and theoretically improve the anchoring of helical blade and provide early stability. A randomized clinical study showed that no patient in the augmented PFNA group had reoperation due to mechanical failure or internal fixation displacement compared to 6 patients with fixation failure in the normal PFNA group [6]. The augmented PFNA may prevent reoperation by strengthening bone structure. Many other studies have produced a similar result that bone cement-augmented PFNA increase the stability of internal fixation and promote postoperative functional recovery without implantation-related complications [37, 38]. However, the number of cases and the follow-up time is relatively limited, the clinical efficacy remain to be further observed.

Finite element method has been widely used in the study of implant biomechanics. Goffin et al. [39] established a model of femoral intertrochanteric fracture without medial support and simulated osteoporosis by changing density, and found that helical blade improved the anchoring force through compressing trabecular bone around it, thus reducing the risk of cutting, especially in the osteoporotic femoral head. Seker et al. [40] modeled different types of intertrochanteric fractures according to AO classification, simulating one- and two-legged standing postures to explore whether early weight bearing was allowed after intramedullary fixation of intertrochanteric fractures. To our best knowledge, there are few finite element study on the biomechanical mechanism of bone cement augmented PFNA in treating intertrochanteric fracture. Bone tissue are nonlinear, inhomogeneous, anisotropic viscoelastic materials and sensitive to strain rate [41]. There is a complex mathematic relation between strain rate and yield property of bone tissue. Hansen et al. [42] conducted longitudinal tests on human femoral cortical bone and obtained the stress-strain curves of human femoral cortical bone at different strain rates under tension and compression. In numerical simulation, the selection of material constitutive model has a very important effect on simulation accuracy of finite element model. In this study, the bilinear elastoplastic material model (MAT_03) in Hypermesh software was used to characterize the mechanical behavior of bone tissue [16], and the Cowper-Symonds model was used for strain rate, which represented the yield stress through parameters related to strain rate. The failure is defined by setting a failure plastic strain in bone and it occurs by element elimination when the plastic strain of an element reach the limit. In this study, AO31-A1.3 and AO31-A2.2 intertrochanteric fractures were chosen as the representative of stable and unstable type. The FEM was used to explore the influence of bone cement strengthening on PFNA treating intertrochanteric fracture.

Our study shows that bone cement enhanced the cutting resistance of PFNA. In unstable intertrochanteric fractures, the ruptured lateral wall fail to provide stable lateral support and counteract rotation of the head-neck fragment [43]. Meanwhile, the broken medial wall with dissociative lesser trochanter are also unable to provide effective medial support when the head-neck fragment varus [44]. Further, most aged people are osteoporotic with unsubstantial trabecular bone which lead to less contact area with helical blade. When stress exceed the maximum that trabecular bone can withstand, trabecular bone around the helical blade collapse, and the varus of the head-neck fragment cause the cutting effect. Compared to AO31-A1.3 model, AO31-A2.2 demonstrated a higher cutting displacement of helical blade with a lower stress of PFNA which confirm that the unstable intertrochanteric fracture was more likely to cutout (Tables 4 and 5). When head-neck fragment varus at 2° (Figs. 3 and 4), compared to non-augmented model, the head-neck trabecular bone of augmented model showed a relatively dispersed stress and a lower maximum stress value, especially, the decrease of maximum stress value in unstable AO31-A2.2 was more significant than that in stable AO31-A1.3 (Table 4). The bone cement allows for both enhanced fixation at the screw-cement and cement-bone interface, potentially from improved infiltration of both the blade and the trabecular bone. Similarly, the overall displacement of femur after bone cement reinforced was lower than that of non-enhanced models (Fig. 7, Table 5), and it seems that the displacement of unstable type femur decreased more significantly indicating that bone cement reinforcement can improve the biomechanical stability of the femur. These changings and differences proved that bone cement-augmented PFNA can resist varus deformity of femoral head in intertrochanteric fractures, especially in unstable fracture.

Compared with the non-augmented PFNA fixation, the augmented PFNA demonstrated a lower stress and displacement of femur, and the unstable fracture type acquire a better biomechanical effect. Stress concentration on helical blade is one of the main causes of damage. Bone cement can effectively increase the stability of internal fixation, disperse and conduct stress to avoid concentration on blade tip and reduce the risk of cutout. The lateral femoral wall in unstable femoral intertrochanteric fractures are incomplete, but the proximal end of a helical blade wrapped in cement directly reinforces the medial support of PFNA, and its reliable stability can partially reduce the impact of incomplete lateral wall on fixation, to some extent against varus of head-neck fragment; The continuity of the posterior and medial femoral neck cortical bone is critical for the mechanical stability of intertrochanteric fractures [45], while the fractured lesser trochanter fail to provide effective support to head-neck fragment resulting in varus deformity [46], but the femoral head is subjected to a less portion of the downward stress since a large of it transfer to PFNA after cement augmented therefore partially reduce the medial support of the lesser trochanter. However, theoretically, cement augmented-PFNA potentially has a fatigue disadvantage compared to non-augmented-PFNA. After cement reinforced, the helical blade is integrated with the head-neck fragment, stresses conducted from femoral head and concentrated at the junction between nail and blade. If the healing of fracture delayed or nonunion, fatigue fracture may still occur in internal fixation under long-term cyclic loading. In fact, there are numerous reports of nail breakage specifically in the new generation nail Trochanteric Femoral Nail Advanced(TFNA), some associated with TFNA cement augmentation [47]. Whilst the decreased lateral wall thickness may implicate the risk of implant failure [48], this FE study provide another possible explanation on the phenomena of the breakage in distal interlock screw or nail-blade conjunction.

This study has few major limitations. The material characteristics in this study were assumed to be homogeneous, isotropic and strain rate dependent to simplify the simulation and were adopted from previous studies. PFNA is superior in treating unstable intertrochanteric fractures partly because the helical blade can compress the osteoporotic trabecular bone in the femoral head and enhance the anchoring, however, the compaction effect was not simulated in this study. Although some simplification has been taken in this study and will differ from actual situations in clinical practice, the topic examined in this study still have clearer trends and results.

## Conclusion

This study investigated the stress and displacement changing in non-cement and cement augmented PFNA in treating two type of intertrochanteric fractures, results support the conclusion that the use of cement augmentation reduce the risk of cutout in both stable and unstable intertrochanteric fractures, especially efficient for the unstable intertrochanteric fracture.

## Data Availability

The datasets used and/or analysed during the current study are available from the corresponding author on reasonable request.

## Abbreviations

FEMs: Finite element models
TAD: Tip Apex Distance
PFNA: Proximal femoral nail anti-rotation
PMMA: Polymethylmethacrylate
Cal-TAD: Calcar referenced Tip Apex Distance
FEA: Finite element analysis
3D: Three dimension
BMD: Bone mineral density
DHS: Dynamic hip screws
TFNA: Trochanteric Femoral Nail Advanced
